# How I do a brain death examination: the tools of the trade

**DOI:** 10.1186/s13054-020-03376-6

**Published:** 2020-11-18

**Authors:** Eelco F. M. Wijdicks

**Affiliations:** Neurosciences Intensive Care Unit, Saint Marys Hospital, Mayo Clinic, 200 First Street SW, Rochester, MN 55905 USA

Brain death has been accepted universally, although practice differences have eluded consensus [1, 2]. Laws and guidelines have not appreciably changed [3, 4] nor have tools of the trade. The following principles remain: establish the reason for coma (most important), exclude known/unknown confounders (equally important), ascertain the futility of intervention (decided before), prepare the patient for testing (to optimize resolution), and acknowledge clinical examination as the benchmark (essential) [5]. One should ask three questions: Have I tried everything to change the clinical picture? Can I proceed? Can I be fooled?

Brain death examination is hands-on (Fig. 1) and focused on brainstem function: from mesencephalon down to the dorsal medulla oblongata. These seemingly few tests are more than sufficient; other tests (e.g., IV atropine, nasal tickle, and ciliospinal reflex) add nothing. In the mesencephalon, test only one reflex circuit, the pupil response to a high-intensity flashlight. Pupils in brain death are not “fixed and dilated” but mid-position (4–6 mm) due to loss of sympathetic and parasympathetic input. I use a magnifying glass while others use a pupilometer; the only difference between them is several thousand dollars. Several reflex circuits are tested in the pons: absent corneal reflexes; squirt water on the cornea or strike with cotton from the conjunctiva toward and on the cornea. (Sadly, one in five surveyed members of professional organizations does not test correctly [6]). To elicit the oculocephalic reflex, hold the head firmly with two hands while keeping the eyelids open with thumbs. Eye movement (opposite to head movement) is induced by fast head turning from a middle portion to 90° on both sides. (Obviously, omit this test in a trauma patient with a cervical collar.) Also, eye movements should be absent after irrigating the tympanum with 30 cc ice water. (The normal response in a comatose patient is a very slow deviation of the eyes toward the syringe.) I place pen marks on the eyelid to reference the level of the pupil. Pain grimaces should be absent upon deep pressure to nail beds (reflex hammer), pressure on the supraorbital nerve (thumb), or deep pressure on the temporomandibular joint condyles (index fingers).

**Fig. 1:**
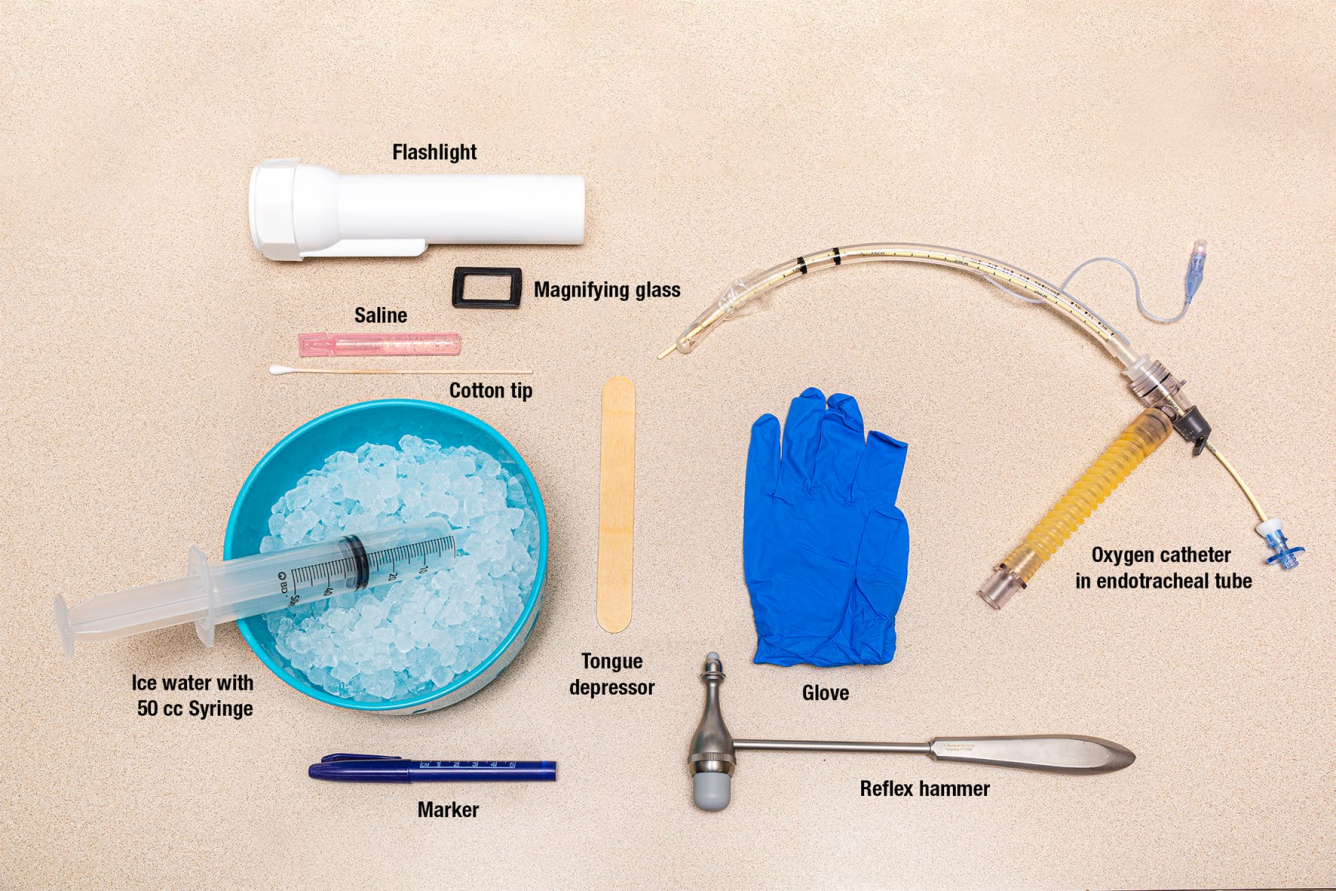
Simple (low-tech) bedside tools needed to perform a brain death examination. Each object has a function (see text for a description). Note the small oxygen insufflation catheter is placed just out of the endotracheal tube, which can be preset in a dummy endotracheal tube before connection to the patient

In the medulla oblongata, test the gag response with a tongue depressor or suction device into the oral cavity. As it is difficult to see, I insert a gloved finger past the uvula, a more reliable stimulus. Catheter passages through the endotracheal tube while providing suctioning pressure should not elicit a cough response.

Noxious stimuli should not produce a motor response. While there might be a spinally mediated response (i.e., brief, slow movements in the upper limbs, flexion in the fingers, or arm lifting), they are never coordinated decerebrate or decorticate responses [3–6] and diminish with repeated stimulation. Plantar reflexes are absent, but upward toe flexion may occur with a triple-flexion response.

Next is the apnea test. Keep it simple [7]. Review the chest X-ray and blood gas and pre-oxygenate to PaO_2_ > 200 mmHg. The factors predicting a problematic apnea test are (1) insufficient pre-oxygenation, (2) high A-a gradient (> 300), (3) oxygen through a T-piece, (4) systolic hypotension (< 90 mmHg), and (5) baseline acidosis (arterial pH < 7.30). Pulmonary edema (or massive infiltrates) produces a significant A-a gradient and failure to oxygenate during the test. In some patients, neurogenic pulmonary edema resolves in 48 h, still allowing an apnea test. Especially germane today, apnea testing is unsafe in COVID-19 pneumonia with neurologic complications due to diffuse exudative epithelial denudation of alveoli. A pretest high PEEP may complicate oxygenation after disconnecting the patient; pretest recruitment maneuver and a 20-cm H_2_O CPAP valve may be used.

Disconnect the ventilator and flow oxygen (6 L/min) through a catheter advanced to the carina (Fig. 1). Monitor oxygen saturation, pulse, and blood pressure while looking for breathing. Breathing occurs quickly (maybe only an early single gasp). When breathing is absent, declare brain death at a pCO_2_ target of 60 mmHg or with a 20 mmHg increase. Conventionally, time of death is the time of the second blood gas result. Complications, usually minor with good preparation, become major with bad or non-standard preparation [7–10]. Our decades-long experience with this oxygen diffusion technique has been safe and aborted in only 3% of 212 tests [9].

ECMO requires adjustment. Blending CO_2_ into the oxygenator is the best option. We estimate that an 8% volume of CO_2_ results in paCO_2_ of 65–70 mmHg. If blending is not available, CO_2_ can only be increased by markedly diminishing sweep gas, but this technique risks hypoxia. Additionally, reducing the sweep gas increases the number of expensive blood gasses; it is anyone's guess where PaCO_2_ will end up [8, 10].

Ultimately, the physician determining brain death must use his own best judgment. Sequential steps are essential (Fig. 2). Whether one absent pontomesencephalic reflex should prompt an ancillary test is debatable, because death comes with loss of medulla oblongata function. Focus on the functionality of the lower brainstem because there is a vertical loss. It is a one-way door. Ancillary (“confirmatory”) tests remain mandated in a minority of countries as a safeguard or when unable to complete the apnea test. But studies of ancillary tests lacked appropriate controls; comparisons between tests show major discrepancies and technical problems (or even timely availability). In several countries, repeated comprehensive evaluations are required. No literature-based evidence supports a second examination contradicting the first. Longer wait times are unsupported by evidence or facts.

**Fig. 2:**
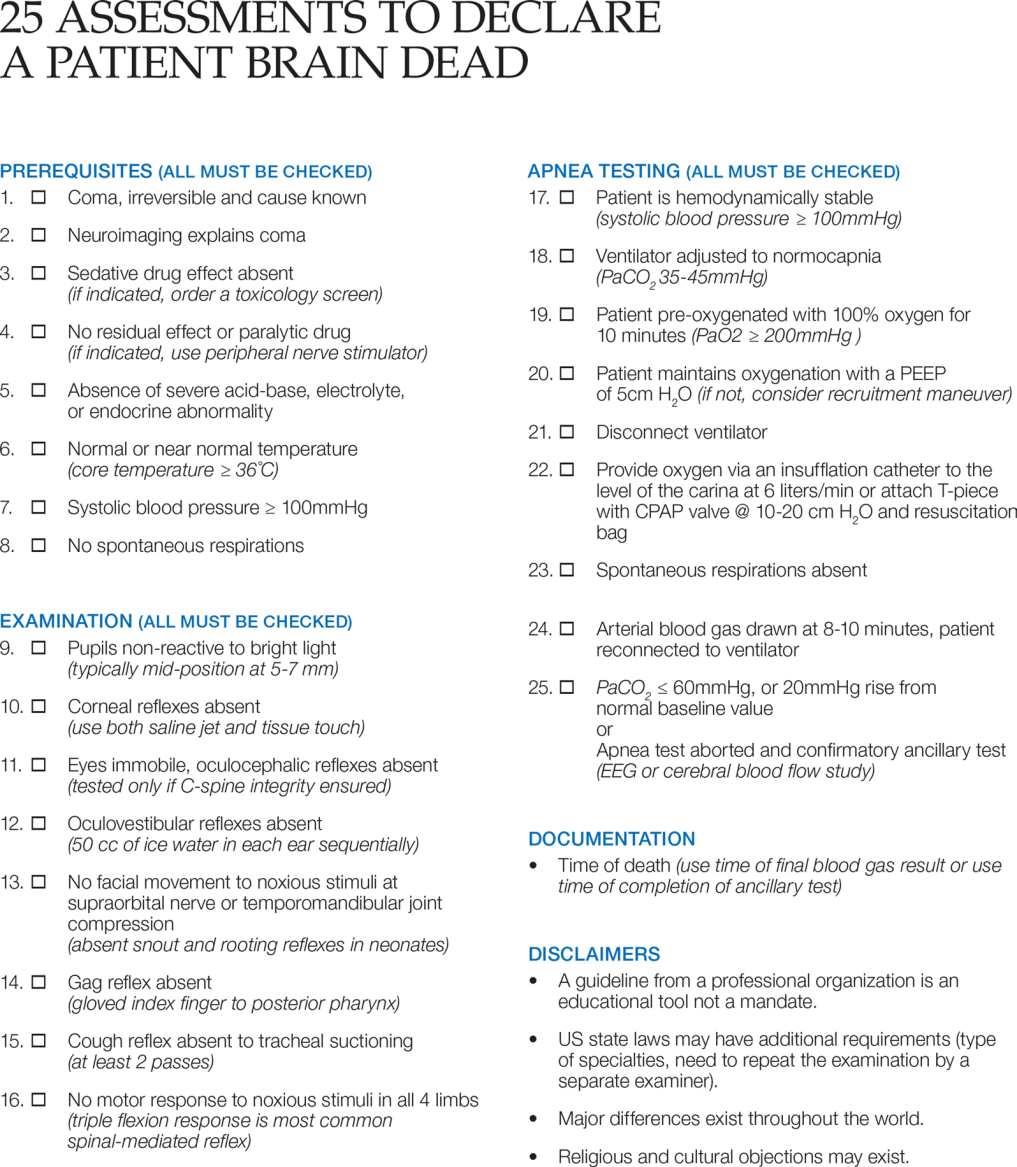
Steps in declaring brain death

Training is warranted but current mannequin-based programs lack validity. Without built-in brainstem reflexes, mannequins cannot simulate spared brainstem reflexes with the notable exception of preserved breathing drive. Simulation is best used to teach the recognition of confounders [11].

Every intensive care physician is able to perform a full clinical brain death examination. Do not resolve clinical uncertainties with an ancillary test with poor specificity. Proceed when you can—with colleagues if they are available. If available, ask to involve a neurointensivist. For now, we must trust the examiner is fully knowledgeable and competent in all aspects.

## Data Availability

All data generated or analyzed during this study are included in this published article.
